# Multivariate Analysis of Correspondence between Left Atrial Volumes Assessed by Echocardiography and 3-Dimensional Electroanatomic Mapping in Patients with Atrial Fibrillation

**DOI:** 10.1371/journal.pone.0152553

**Published:** 2016-03-29

**Authors:** Stepan Havranek, Martin Fiala, Alan Bulava, Libor Sknouril, Miroslav Dorda, Veronika Bulkova, Zdenka Fingrova, Lucie Souckova, Tomas Palecek, Jan Simek, Ales Linhart, Dan Wichterle

**Affiliations:** 1 2nd Department of Medicine - Department of Cardiovascular Medicine, First Faculty of Medicine, Charles University and General University Hospital in Prague, Prague, Czech Republic; 2 Department of Cardiology, Heart Centre, Hospital Podlesi, Trinec, Czech Republic; 3 Department of Internal Medicine and Cardiology, University Hospital, Masaryk University, Brno, Czech Republic; 4 Department of Cardiology, Hospital Ceske Budejovice and Faculty of Health and Social Studies, University of South Bohemia, Ceske Budejovice, Czech Republic; 5 Department of Cardiology and Angiology, St. Anne’s University Hospital and International Clinical Research Center, Brno, Czech Republic; 6 Department of Cardiology, Institute for Clinical and Experimental Medicine, Prague, Czech Republic; Harvard Medical School, UNITED STATES

## Abstract

**Background:**

Left atrial (LA) enlargement is a predictor of worse outcome after catheter ablation for atrial fibrillation (AF). Widely used two-dimensional (2D)-echocardiography is inaccurate and underestimates real LA volume (LAV). We hypothesized that baseline clinical characteristics of patients can be used to adjust 2D-ECHO indices of LAV in order to minimize this disagreement.

**Methods:**

The study enrolled 535 patients (59 ± 9 years; 67% males; 43% paroxysmal AF) who underwent catheter ablation for AF in three specialized centers. We investigated multivariately the relationship between 2D-echocardiographic indices of LA size, specifically LA diameter in M-mode in the parasternal long-axis view (LAD), LAV assessed by the prolate-ellipsoid method (LAV_Ellipsoid_), LAV by the planimetric method (LAV_Planimetry_), and LAV derived from 3D-electroanatomic mapping (LAV_CARTO_).

**Results:**

Cubed LAD of 106 ± 45 ml, LAV_Ellipsoid_ of 72 ± 24 ml and LAV_Planimetry_ of 88 ± 30 ml correlated only modestly (r = 0.60, 0.69, and 0.53, respectively) with LAV_CARTO_ of 137 ± 46 ml, which was significantly underestimated with a bias (±1.96 standard deviation) of -31 (-111; +49) ml, -64 (-132; +2) ml, and -49 (-125; +27) ml, respectively; p < 0.0001 for their mutual difference. LA enlargement itself, age, gender, type of AF, and the presence of structural heart disease were independent confounders of measurement error of 2D-echocardiographic LAV.

**Conclusion:**

Accuracy and precision of all 2D-echocardiographic LAV indices are poor. Their agreement with true LAV can be significantly improved by multivariate adjustment to clinical characteristics of patients.

## Introduction

Catheter ablation for atrial fibrillation (AF) is an established therapy in selected patients [1]. Assessment of left atrial (LA) size, which has been identified as a predictor of catheter ablation efficacy [2, 3], is essential when this treatment is considered. Despite advances in quantification of LA anatomy, the simplest echocardiographic index—antero-posterior LA diameter (LAD) from parasternal long-axis view—has been predominantly used for risk stratification of AF recurrence in numerous ablation studies as reflected by a recent meta-analysis [4].

It has long been known, however, that LAD poorly correlates with LA volume (LAV) [5–8], which has lead to the introduction of various complex methods for the calculation of LAV by use of 2D-echocardiography (ECHO) (e.g. prolate-ellipsoid method, area-length or disc method in single or biplane modification) [5–9]. While providing a more accurate assessment of LA size than LAD [5–9], they still systematically underestimate LAV assessed by 3D-ECHO, CT or MRI [7–12]. There is limited data on confounders of inaccuracy of 2D-ECHO indices. To the best of our knowledge, only single study reported LA enlargement to be associated with poor correspondence between LA diameters and 3D-ECHO LAV [8].

We hypothesized that other simple clinical characteristics of patients influencing this discrepancy could be identified in larger population and subsequently used for appropriate adjustment of 2D-ECHO indices. We investigated this hypothesis in real-world population of patients with non-valvular AF scheduled for catheter ablation in whom electroanatomic 3D reconstruction of the LA can be performed [13] and LAV can be assessed without geometric assumptions [14, 15].

## Methods

### Patients

Consecutive patients, who underwent catheter ablation for AF at three cardiology centers between May 2007 and December 2013, were analyzed. The data were retrieved from a dedicated registry that was shared by the centers. The study was approved by the local ethics committees at all three institutions involved (General University Hospital in Prague, Hospital Ceske Budejovice, Hospital Podlesi in Trinec) and all patients gave written informed consent.

### 3D Mapping and CT Image Integration

LA mapping was performed in standardized way prior to the ablation procedure. A 3D electroanatomic mapping system (CARTO XP or CARTO 3, Biosense-Webster Inc., Diamond Bar, CA, USA) and manual catheter navigation was used for reconstruction of the LA endocardial surface. Uniformly distributed mapping points were acquired at sites with stable endocardial contact. Special attention was paid not to include mapping points behind the pulmonary vein ostia. The orifice and proximal part of LA appendage was always mapped. Precise delineation of the mitral annulus was performed in all cases. Intracardiac echocardiography was used to visualize and tag the critical structures. A 3D virtual shell of the LA was built by software interpolations over the co-ordinates of multiple endocardial points. When multi-detector CT reconstruction of LA was available, the CT image was registered to the CARTO map by an algorithm that minimizes the distance between the mapping points and the surface of CT image. A merged display of the CT image and electroanatomic map was used to eliminate incidental internalized and/or externalized mapping points in order to improve the quality of integration. Finally, LAV_CARTO_ was assessed using a built-in computation function of the Biosense system.

### Echocardiographic examination

Transthoracic echocardiographic examinations were performed prior to the ablation procedure according to the recommendations of American Society of Echocardiography [6, 7, 16]. In case of irregular rhythm, the echocardiographic parameters were measured over ten beats to avoid bias given by beat-to-beat variability. The LAD was defined as end-systolic, M-mode, antero-posterior linear dimension in the parasternal long-axis view using 2D guidance for positioning of the cursor. The measurement was cubed (LAD^3^) in order to be comparable to other volume measures. The LAV_Ellipsoid_ was assessed by the prolate-ellipsoid method, which requires three LA orthogonal diameters in end-systole (LAD and two diameters in the apical 4-chamber view). A standardized planimetric method in a single-plane (apical 4-chamber view) was used to obtain LAV_Planimetry_.

### Statistical analysis

Continuous variables were expressed as means with standard deviations and compared by the 2-tailed t-test for independent samples. Categorical variables were expressed as percentages and compared by χ2–test. Pearson’s correlation and multivariate linear regression were used to analyze the relationship between LAD^3^, LAV_Ellipsoid_, and LAV_Planimetry_ (together with other clinical covariates) as independent variables and LAV_CARTO_ as dependent variable. Obtained regression coefficients were used for simple and multivariate adjustment of individual ECHO-based indices. Predictive characteristics of 2D-ECHO-based LAV for above-median LAV_CARTO_ were assessed for raw echocardiographic measurements as well as for the values after multivariate adjustment for the clinical characteristics of patients. A p-value < 0.05 was considered significant. All analyses were performed using the STATISTICA vers.12 software (Statsoft, Inc., Tulsa, USA).

## Results

**Fig 1:**
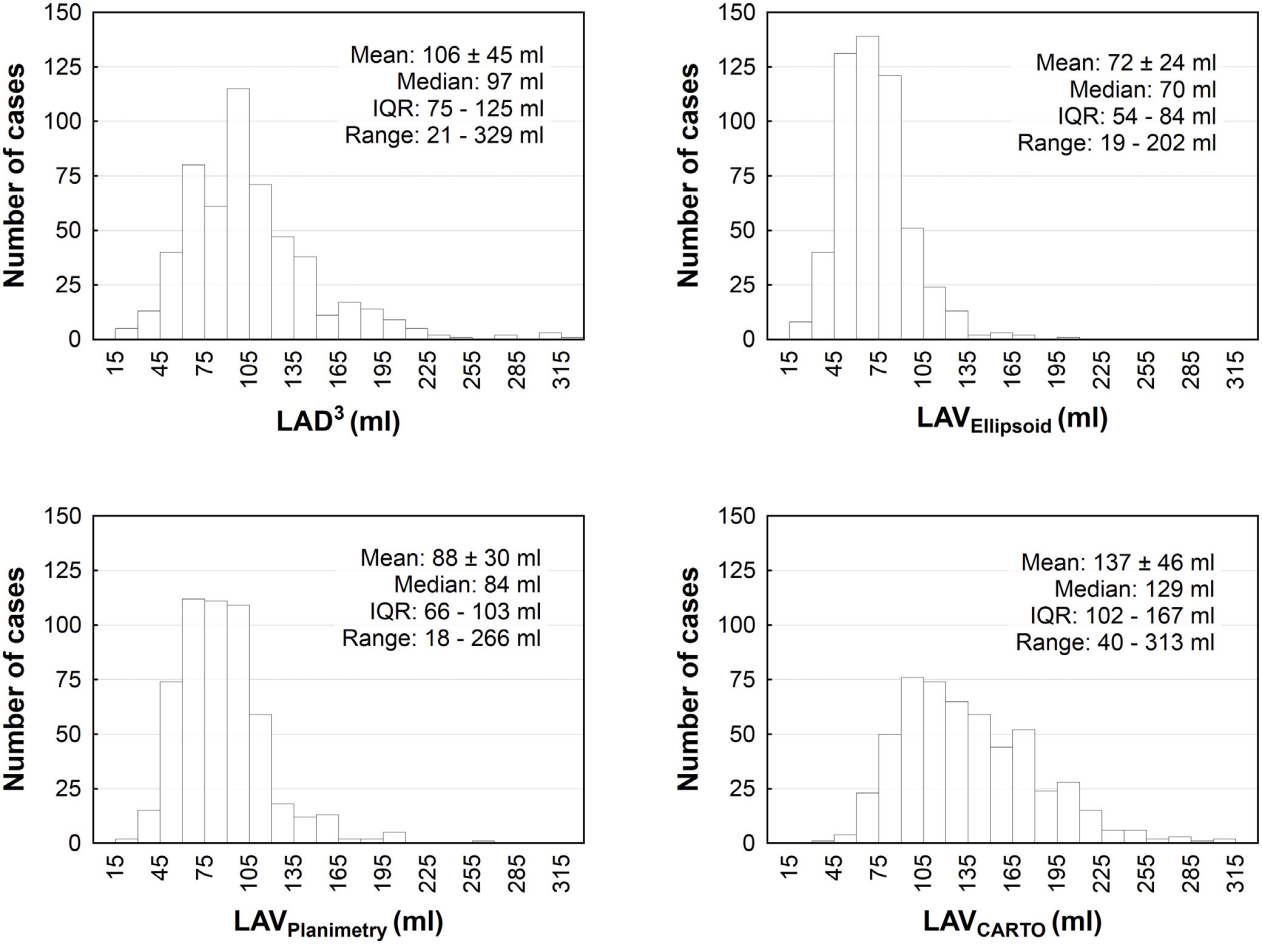
Distribution of left atrial atrial size indices. Abbreviations: IQR—interquartile range; LAD^3^ –cubed left atrial diameter in parasternal long-axis view; LAV_Ellipsoid_−left atrial volume (LAV) assessed by the prolate-ellipsoid method; LAV_Planimetry_−LAV assessed by the planimetric method; LAV_CARTO_−CARTO-derived LAV.

A total 535 patients (aged 59 ± 9 years; 67% males; 43% paroxysmal AF) were analyzed. Baseline characteristics of the total population and subgroups by type of AF are shown in Table 1. Males were significantly younger than females (58 ± 10 vs. 62 ± 8 years, p < 0.0001). The distributions of LAV indices are illustrated in Fig 1.

**Table 1:** Baseline characteristics.

	Total population (n = 535)	Paroxysmal AF (n = 230)	Non-paroxysmal AF (n = 305)	p value
Age (years)	59 ± 9	59 ± 10	60 ± 9	0.60
Males	358 (67%)	141 (61%)	217 (71%)	0.02
Hypertension	335 (63%)	128 (56%)	207 (68%)	0.005
Diabetes mellitus	74 (14%)	30 (13%)	44 (14%)	0.74
Structural heart disease	112 (21%)	22 (10%)	90 (30%)	< 0.0001
Coronary artery disease	45 (8%)	16 (7%)	29 (10%)	0.22
CHADS_2_	1.01 ± 0.87	0.83 ± 0.83	1.15 ± 0.89	< 0.0001
CHA_2_DS_2_-VASc	1.71 ± 1.33	1.59 ± 1.32	1.79 ± 1.33	0.07
LV EF (%)	61 ± 10	64 ± 8	57 ± 11	< 0.0001
LAD (mm)	46 ± 6	44 ± 6	48 ± 6	< 0.0001
LAD^3^ (ml)	106 ± 45	89 ± 37	119 ± 45	< 0.0001
LAV_Ellipsoid_ (ml)	72 ± 24	62 ± 20	79 ± 24	< 0.0001
LAV_Planimetry_ (ml)	88 ± 30	81 ± 29	92 ± 30	< 0.0001
CARTO mapping points	190 ± 65	177 ± 60	201 ± 67	< 0.0001
CT image integration	408 (76%)	168 (73%)	240 (79%)	0.11
LAV_CARTO_ (ml)	137 ± 46	107 ± 32	159 ± 43	< 0.0001

AF—atrial fibrillation; CT—computer tomography; LAD—left atrial diameter in parasternal long-axis view; LAD^3^ –cubed LAD; LAV_Ellipsoid_−left atrial volume (LAV) assessed by the ellipsoid model; LAV_Planimetry_−LAV assessed by the planimetric method; LAV_CARTO_−CARTO-derived LAV; LV EF—left ventricular ejection fraction. Values represent mean ± standard deviation or number (percentage).

**Fig 2:**
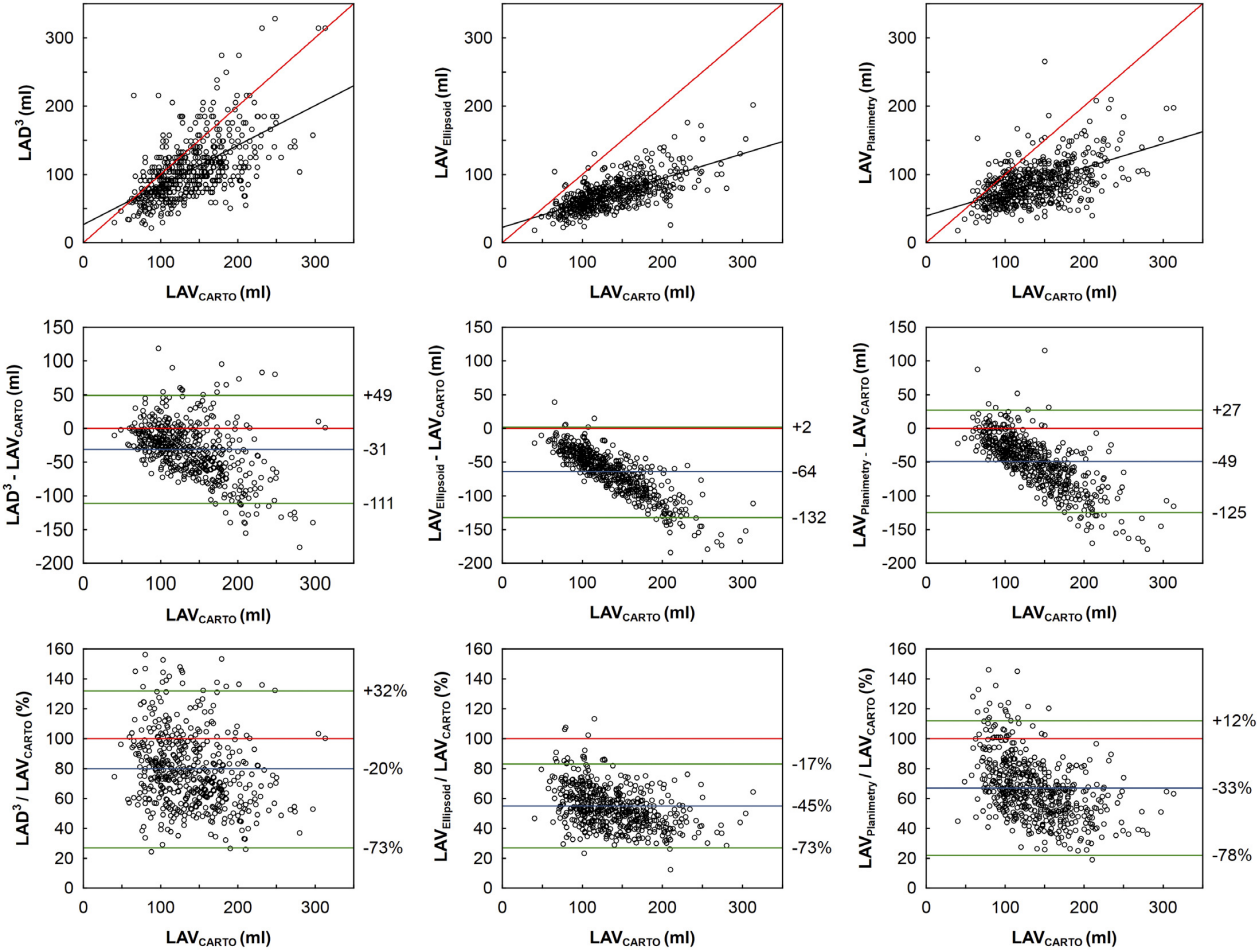
Correlation and agreement between 2D-ECHO-based and CARTO-derived LAV. Upper row: Pearson’s correlation. Middle row: scatterplots for absolute differences between 2D-ECHO-based and CARTO-derived LAV versus CARTO-derived LAV. Lower row: scatterplots for relative differences between 2D-ECHO-based and CARTO-derived LAV versus CARTO-derived LAV. Red line—identity line; black line—regression line; blue line—bias; green line—limits of agreement (±1.96 standard deviation). Abbreviations as in Fig 1.

**Fig 3:**
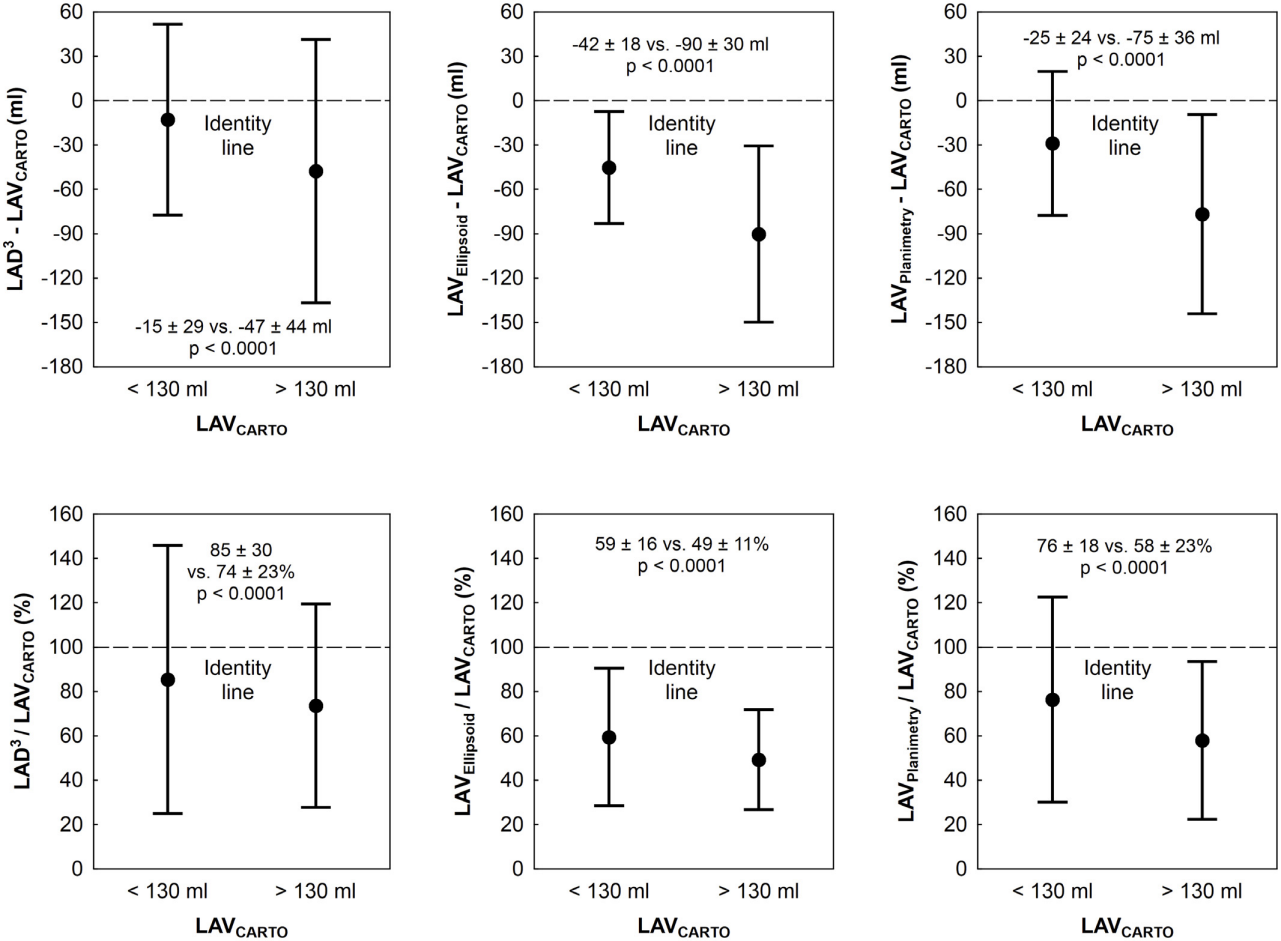
Absolute and relative agreement between 2D-ECHO-based and CARTO-derived LAV in categories by LA size. Absolute and relative differences are shown in upper and lower row of graphs, respectively. Data are dichotomized by median of LAV_CARTO_ (130 ml). The points and whiskers represent mean and ±1.96 standard deviation. Abbreviations as in Fig 1.

The results of simple regression between 2D-ECHO-based LAV indices and LAV_CARTO_ are shown in Table 2 and Fig 2. The differences between correlation coefficients were significant (p < 0.05). LAD^3^, LAV_Ellipsoid_, and LAV_Planimetry_ underestimated LAV_CARTO_ with an absolute bias (± 1.96 standard deviation) of -31 (-111; +49) ml, -64 (-132; +2) ml, and -49 (-125; +27) ml, and a relative bias of -20% (-73%; +32%), -45% (-73%; -17%), and -33% (-78%; +12%), respectively; p < 0.0001 for their mutual difference (Fig 2). For all 2D-ECHO-based LAV indices, the underestimation was significantly (p < 0.0001) more pronounced in patients with above-median (>130 ml) LAV_CARTO_ (Fig 3).

**Table 2:** Univariate regression between individual 2D-ECHO-based LAV indices and LAV_CARTO_ (dependent variable).

LA size parameter	LAD^3^	LAV_Ellipsoid_	LAV_Planimetry_
Correlation coefficient	0.60	0.69	0.53
p value	<0.0001	<0.0001	<0.0001
Intercept (95% CI)	68 (61; 74)	42 (35; 49)	66 (56; 76)
Regression coefficient (95% CI)	0.62 (0.56; 0.68)	1.32 (1.22; 1.42)	0.81 (0.70; 0.92)

CI—confidence interval; other abbreviations as in Table 1.

**Fig 4:**
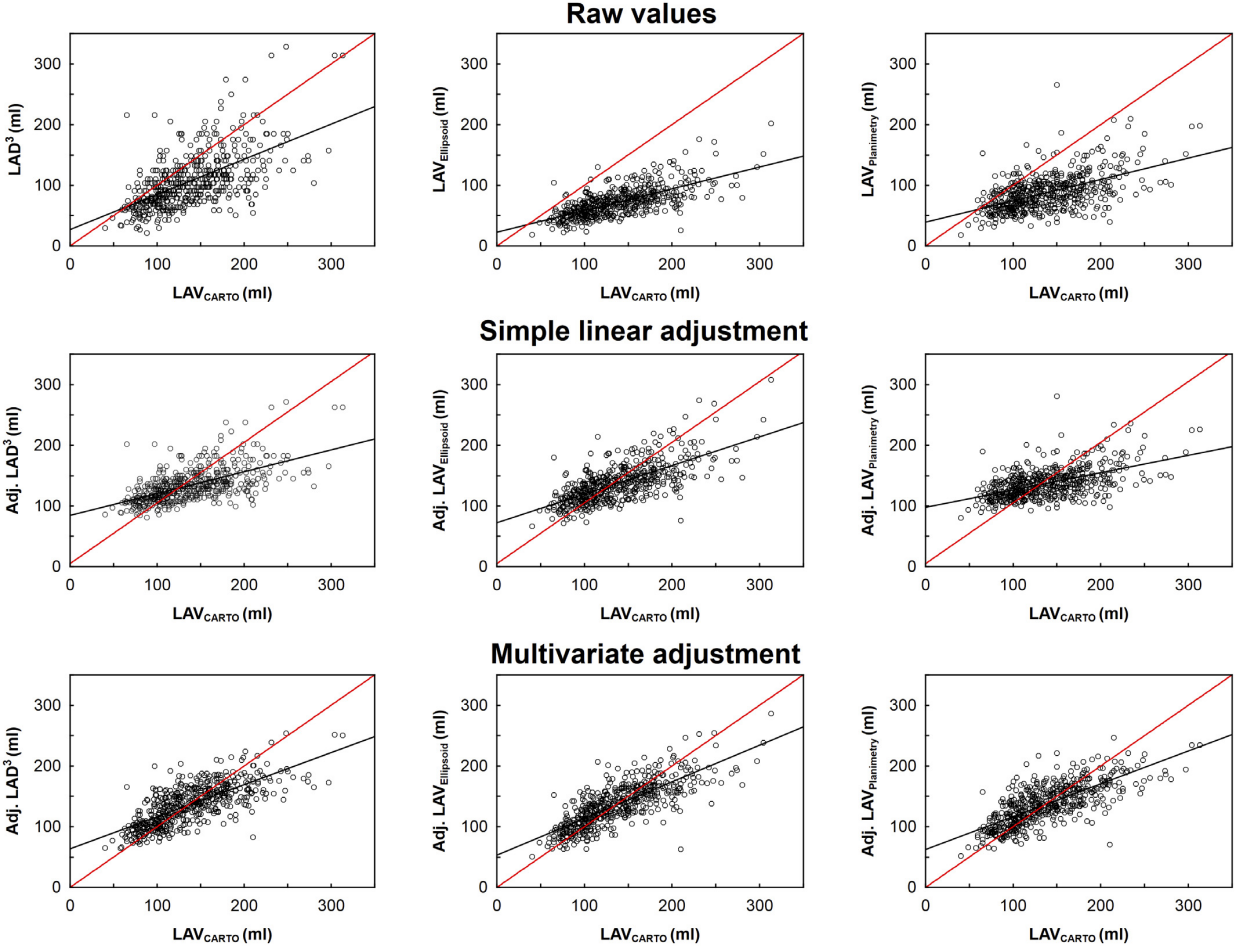
Correlation of raw and adjusted 2D-ECHO-based LAV with CARTO-derived LAV. Upper row: raw values. Middle row: simple linear adjustment. Lower row: multivariate adjustment for clinical covariates. Regression coefficients from Tables 2 and 3 were used for the simple and multivariate adjustment, respectively. Specifically, adjusted LAV was calculated as: 68 + 0.62 LAD^3^; 42 + 1.32 LAV_Ellipsoid_; 66 + 0.81 LAV_Planimetry_; 67 + 0.42 LAD^3^ + 15 (if male) + 0.44 Age + 34 (if persistent AF) + 14 (if SHD); 49 + 0.98 LAV_Ellipsoid_ + 12 (if male) + 0.34 Age + 32 (if persistent AF) + 10 (if SHD); 64 + 0.60 LAV_Planimetry_ + 18 (if male) + 0.38 Age + 40 (if persistent AF) + 14 (if SHD). Red line–identity line; black line–regression line. Adj = adjusted. Other abbreviations as in Fig 1.

Age, gender, type of AF, and presence of structural heart disease (SHD) were significant and independent covariates of the difference between 2D-ECHO-based and CARTO-derived LAV by multivariate regression analysis (Table 3). The most pronounced underestimation was found in elderly males with dilated LA, non-paroxysmal AF, and SHD. Overestimation was quite rare and limited namely to young females with non-dilated LA, paroxysmal AF, and absence of SHD. The Fig 4 shows how 2D-ECHO-based LAV indices correspond to CARTO-derived LAV after simple linear and multivariate adjustment.

**Table 3:** Stepwise forward multivariate regression analysis of determinants of LAV_CARTO_.

	Model A LA size = LAD^3^ (R = 0.74, P<0.0001)		Model B LA size = LAV_Ellipsoid_ (R = 0.78, P<0.0001)		Model C LA size = LAV_Planimetry_ (R = 0.74, P<0.0001)	
	Regression coefficient (95% CI)	p value	Regression coefficient (95% CI)	p value	Regression coefficient (95% CI)	p value
Intercept	67 (47; 87)	< 0.0001	49 (30; 68)	< 0.0001	64 (44; 84)	< 0.0001
LA size (ml)	0.42 (0.36; 0.49)	< 0.0001	0.98 (0.87; 1.10)	< 0.0001	0.60 (0.51; 0.69)	< 0.0001
Male gender (YES = 1 / NO = 0)	15 (9; 21)	< 0.0001	12 (7; 18)	< 0.0001	18 (12; 24)	< 0.0001
Age (years)	0.44 (0.14; 0.74)	0.004	0.34 (0.06; 0.61)	0.02	0.38 (0.08; 0.68)	0.01
Non-paroxysmal AF (YES = 1 / NO = 0)	34 (28; 40)	< 0.0001	32 (26; 37)	< 0.0001	40 (35; 46)	< 0.0001
Structural heart disease (YES = 1 / NO = 0)	14 (8; 21)	< 0.001	10 (4; 17)	0.001	14 (7; 20)	< 0.0001

LA—left atrium; CI—confidence interval; other abbreviations as in Table 1.

**Fig 5:**
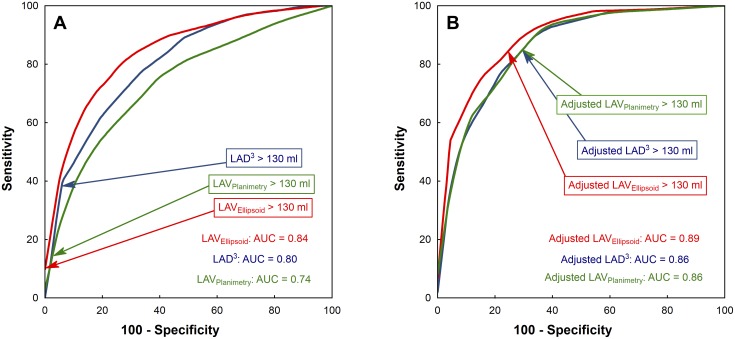
Receiver operating characteristics (ROC) for the prediction of CARTO-derived LAV > 130 ml. ROC curves are plotted for LAD^3^ (in blue), LAV_Ellipsoid_ (in red) and (3) LAV_Planimetry_ (in green). Panel A: ROC for raw 2D-ECHO-based LAV. Panel B: ROC for multivariately adjusted 2D-ECHO-based LAV (the adjustment was the same as in Fig 4). Points corresponding to 2D-ECHO-based LAV > 130 ml are indicated by arrows. AUC—area under the curve; other abbreviations as in Fig 1.

The poor ability of LAD^3^, LAV_Ellipsoid_, and LAV_Planimetry_ to predict LAV_CARTO_ > 130 ml is demonstrated by receiver operating characteristics with mutually different (p < 0.01) areas under the curve (AUC) of 0.80, 0.84, and 0.74, respectively (Fig 5A). Predictive power was improved only modestly (AUC = 0.86, 0.89, and 0.86) after adjustment for clinical covariates (Fig 5B). The AUC for LAV_Ellipsoid_ remained the highest and differed significantly from those for LAD^3^ and LAV_Planimetry_ (p < 0.01 for both comparisons).

## Discussion

The study performed in a relatively large, real-world population of patients with non-valvular AF confirmed substantial disagreement between LAV assessed by 2D-ECHO methods and reference LAV obtained by 3D electroanatomic mapping. The major finding of the study is that this disagreement was influenced by LA enlargement itself, gender, age, type of AF, and presence of SHD. Moreover, the impact of these factors was consistent for all 2D-ECHO-based LAV indices. Corresponding adjustment of echocardiographic indices resulted in improvement of their accuracy and precision. LAV_Ellipsoid_ had the largest systematic deviation from LAV_CARTO_ but offered the narrowest limits of agreement and, consequently, provided the best concordance with LAV_CARTO_ either after simple or multivariate adjustment.

The disagreement between LA size measures by different methods is not a novel observation. As CT, MRI or 3D-ECHO has improved understanding of the LA as an asymmetrically shaped 3D structure [9–12], the sphericity assumption in echocardiographic measurements results in underestimation of true the LAV by M-mode or 2D-ECHO estimates of LA size [6–8, 11], especially when atria are enlarged [8]. Apart from dilatation, atrial remodelling in patients with persistent AF or SHD is associated with a change of LA shape, which renders the standard geometric models even more inadequate. Such changes include, for example, a trapezoidal LA shape [17], enlargement of the pulmonary vein antrum dimension [18], LA roof re-shaping [19], dilatation of the anterior part of the LA [20], and spherical deformation of the LA [21]. Age and gender may also play a role in the degree of atrial remodelling leading to inaccuracy of standard geometric models. For example, the LAD measured in M-mode progressively increased with age, but was not followed by a change in echocardiographically assessed LAV until the age of 80 years [22], providing evidence that the LA changes shape during life [23]. The LAD was smaller in women than in men, but sex-related differences in any indices of LA morphology [22] or indexed LAV [23] were not identified.

It could be expected that the lack of constant proportion between the major axes in remodelled and enlarged LA more likely affects LA size parameters based on single-dimensional measurement, i.e. M-mode antero-posterior diameter. Our data, however, showed that none of analyzed parameters was associated with clearly superior agreement with CARTO-derived LAV. Although the prolate-ellipsoid method based on composition of three linear dimensions provided modestly higher precision (better correlation with LAV_CARTO_ and, consequently, better prediction of LA enlargement) than LAV_Planimetry_ and LAD^3^, the LAV_Ellipsoid_ demonstrated the lowest accuracy (the largest underestimation) of LAV_CARTO_. This is in agreement with previous studies systematically reporting smaller LAV assessed by the prolate-ellipsoid method compared with 2D-ECHO planimetry or 3D-ECHO [8, 22, 24]. The novel chamber quantification guidelines discuss different methods available for measurement of LA volumes [7]. The document recommends the disk summation algorithm for LA size estimation, which was not used in our study. The biplane area-length method provided small additional accuracy compared to the single plane method in a 3D-ECHO study [8]. Moreover, strong correlations and negligible bias were found between LAV measured by single-plane area-length method in the apical 4-chamber view and the biplane method (r = 0.97) [5]. Single-plane apical four chamber LA volumes were smaller than biplane volumes by 2 to 4 mL only [7]. Even the well-validated biplane 2D-based methods systematically underestimated LAV when compared with 3D-ECHO, MRI or CT [11, 12]. We therefore speculate that biplane LAV assessment would not likely improve the correspondence between 2D-ECHO indices and CARTO-derived LAV in our study significantly. Previous study reported excellent correlation (r = 0.9) between CARTO-derived and the biplane disc-method LAV which was systematically smaller (by 20–30%) than 2D-ECHO [14]. This was, however, a single center study in a relatively small number of patients with paroxysmal AF only. Our three-center study included a larger population with clinical characteristics better corresponding to contemporary patients undergoing catheter ablation for AF; in particular, our patients had a considerably wider range of LA size. It is likely that not only different methods for 2D-ECHO LAV assessment, but also dissimilar study design are responsible for the differences between the results of this and our study.

We considered a CARTO-derived LAV instead of CT as the reference LAV measure in our study because CT imaging was not available in 24% of patients. High level of correspondence between LA CARTO map and CT-assessed LA anatomy was already demonstrated [13]. LAV assessment by electroanatomic mapping has also been shown to have reasonable agreement with LAV assessed by intra-procedural 3D cone-beam CT angiogram [25] in one small study. In our study, high-density, point-by point electroanatomic maps were created by experienced operators. When CT image integration was performed (76%), it invariably exhibited excellent spatial agreement between CARTO maps and CT images. This warrants the utility of 3D-mapping-derived LAV as a reference measure. In case of high-density 3D electroanatomic mapping, individual inaccuracies in the location of individual points due to respiration or irregular heart rhythm are mutually cancelled.

On the other hand, echocardiographic assessment is patient-dependent (for example, poor window) and observer-dependent, with the need for appropriate angulation and gain adjustment for clear visualization of the LA endocardium including pulmonary vein confluences and the LA appendage, proper end-systolic timing for diameter measurement and correct identification of the long axis view [5, 24]. For asymmetrical structures, the feasible view for the diameter or area reading does not necessarily correspond to the optimum plane. Limited resolution of 2D-ECHO, precision of border detection and the ability to include trabeculae might contribute to low accuracy of 2D-ECHO in comparison to 3D methods. For all of these reasons, we believe that the disagreement between methods of LAV assessment is predominantly due to inherent inaccuracy of 2D-ECHO methods.

The 3D-echocardiography was not easily available at the time when data collection for this study had been initiated. Such advanced echocardiographic technology might improve the accuracy of LAV readings. Despite advantages in 3D-ECHO, however, the lack of a standardized methodology and limited normative data prevent 3D-ECHO from the routine use for LA size quantification [7]. Moreover, an underestimation of CARTO-derived LAV by 3D-ECHO has been also demonstrated [15].

### Study limitations

The study has several limitations. First, it was not prospectively designed and the data collection was not independently monitored. Second, centers did not contribute equally to the total study population and some imbalance in patients characteristics also appeared among centers. Third, biplane-disc methods were not used in our study, so we could not analyze the potential benefit from biplane compared to single plane assessment of LAV in terms of accuracy and precision. Fourth, proposed equations for adjustment of ECHO-based LAV were not validated in independent population. Finally, the results cannot be probably translated to general population of cardiac patients as well as to patients with valvular AF.

### Conclusions

The substantial disagreement between 2D-ECHO-based LAV and LAV obtained by 3D electroanatomic mapping in patients with non-valvular AF was confirmed. The disagreement can be attributed to both non-spherical LA shape and echocardiographic measurement error. Inaccuracy of 2D-ECHO-based LAV is predominantly driven by the magnitude of LA enlargement. Precision can be improved by adjustment for simple clinical covariates. Because considerable disagreement still exists even after multivariate confounders are taken into account, the relevance of 2D-ECHO-based LA size indices for selection of suitable catheter ablation candidates should be considered with caution. Especially in patients with dilated LA, unadjusted 2D-ECHO-based LAV significantly underestimated the true LAV.

## References

[1] Calkins H, Kuck KH, Cappato R, Brugada J, Camm AJ, Chen SA, et al 2012 HRS/EHRA/ECAS Expert Consensus Statement on Catheter and Surgical Ablation of Atrial Fibrillation: recommendations for patient selection, procedural techniques, patient management and follow-up, definitions, endpoints, and research trial design. Europace. 2012;14:528–606. 10.1093/europace/eus027 22389422

[2] Berruezo A, Tamborero D, Mont L, Benito B, Tolosana JM, Sitges M, et al Pre-procedural predictors of atrial fibrillation recurrence after circumferential pulmonary vein ablation. Eur Heart J. 2007;28:836–41. 17395676 10.1093/eurheartj/ehm027

[3] von Bary C, Dornia C, Eissnert C, Nedios S, Roser M, Hamer OW, et al Predictive value of left atrial volume measured by non-invasive cardiac imaging in the treatment of paroxysmal atrial fibrillation. J Interv Card Electrophysiol. 2012;34:181–8. 10.1007/s10840-011-9641-6 22228410

[4] Zhuang J, Wang Y, Tang K, Li X, Peng W, Liang C, et al Association between left atrial size and atrial fibrillation recurrence after single circumferential pulmonary vein isolation: a systematic review and meta-analysis of observational studies. Europace. 2012;14:638–45. 10.1093/europace/eur364 22117033

[5] Lester SJ, Ryan EW, Schiller NB, Foster E. Best method in clinical practice and in research studies to determine left atrial size. Am J Cardiol. 1999;84:829–32. 10513783 10.1016/s0002-9149(99)00446-4

[6] Lang RM, Bierig M, Devereux RB, Flachskampf FA, Foster E, Pellikka PA, et al Chamber Quantification Writing Group; American Society of Echocardiography's Guidelines and Standards Committee; European Association of Echocardiography. Recommendations for chamber quantification: a report from the American Society of Echocardiography's Guidelines and Standards Committee and the Chamber Quantification Writing Group, developed in conjunction with the European Association of Echocardiography, a branch of the European Society of Cardiology. J Am Soc Echocardiogr. 2005;18:1440–63. 16376782 10.1016/j.echo.2005.10.005

[7] Lang RM, Badano LP, Mor-Avi V, Afilalo J, Armstrong A, Ernande L, et al Recommendations for cardiac chamber quantification by echocardiography in adults: an update from the American Society of Echocardiography and the European Association of Cardiovascular Imaging. J Am Soc Echocardiogr. 2015;28:1–39. 10.1016/j.echo.2014.10.003 25559473

[8] Badano LP, Pezzutto N, Marinigh R, Cinello M, Nucifora G, Pavoni D, et al How many patients would be misclassified using M-mode and two-dimensional estimates of left atrial size instead of left atrial volume? A three-dimensional echocardiographic study. J Cardiovasc Med. 2008;9:476–84. 10.2459/JCM.0b013e3282f194f0 18403999

[9] Hof I, Arbab-Zadeh A, Scherr D, Chilukuri K, Dalal D, Abraham T, et al Correlation of left atrial diameter by echocardiography and left atrial volume by computed tomography. J Cardiovasc Electrophysiol. 2009;20:159–63. 10.1111/j.1540-8167.2008.01310.x 19220573

[10] Vandenberg BF, Weiss RM, Kinzey J, Acker M, Stark CA, Stanford W, et al Comparison of left atrial volume by two-dimensional echocardiography and cine-computed tomography. Am J Cardiol. 1995;75:754–7. 7900683 10.1016/s0002-9149(99)80676-6

[11] Rodevan O, Bjornerheim R, Ljosland M, Maehle J, Smith HJ, Ihlen H. Left atrial volumes assessed by three- and two-dimensional echocardiography compared to MRI estimates.1999;15:397–410. 10.1023/a:1006276513186 10595406

[12] Mor-Avi V, Yodwut C, Jenkins C, Kuhl H, Nesser HJ, Marwick TH, et al Real-time 3D echocardiographic quantification of left atrial volume: multicenter study for validation with CMR. JACC Cardiovasc Imaging. 2012;5:769–77. 10.1016/j.jcmg.2012.05.011 22897989

[13] Piorkowski C, Hindricks G, Schreiber D, Tanner H, Weise W, Koch A, et al Electroanatomic reconstruction of the left atrium, pulmonary veins, and esophagus compared with the "true anatomy" on multislice computed tomography in patients undergoing catheter ablation of atrial fibrillation. Heart Rhythm. 2006;3:317–27. 16500305 10.1016/j.hrthm.2005.11.027

[14] Patel VV, Ren JF, Jeffery ME, Plappert TJ, John Sutton MG St, Marchlinski FE. Comparison of left atrial volume assessed by magnetic endocardial catheter mapping versus transthoracic echocardiography. Am J Cardiol. 2003;91:351–4. 12565098 10.1016/s0002-9149(02)03169-7

[15] Müller H, Burri H, Gentil P, Lerch R, Shah D. Measurement of left atrial volume in patients undergoing ablation for atrial fibrillation: comparison of angiography and electro-anatomic (CARTO) mapping with real-time three-dimensional echocardiography. Europace. 2010;12:792–7. 10.1093/europace/euq031 20185485

[16] Douglas PS, Garcia MJ, Haines DE, Lai WW, Manning WJ, Patel AR, et al ACCF/ASE/AHA/ASNC/HFSA/HRS/SCAI/SCCM/SCCT/SCMR 2011 Appropriate Use Criteria for Echocardiography. J Am Soc Echocardiogr. 2011;24:229–67. 10.1016/j.echo.2010.12.008 21338862

[17] Cozma D, Popescu BA, Lighezan D, Lucian P, Mornos C, Ginghina C, et al Left atrial remodeling: assessment of size and shape to detect vulnerability to atrial fibrillation. Pacing Clin Electrophysiol.2007;30:S147–50. 17302693 10.1111/j.1540-8159.2007.00626.x

[18] Tsao HM, Yu WC, Cheng HC, Wu MH, Tai CT, Lin WS, et al Pulmonary veindilation in patients with atrial fibrillation: detection by magnetic resonance imaging. J Cardiovasc Electrophysiol. 2001;12:809–13. 11469433 10.1046/j.1540-8167.2001.00809.x

[19] Kurotobi T, Iwakura K, Inoue K, Kimura R, Toyoshima Y, Ito N, et al The significance of the shape of the left atrial roof as a novel index for determining the electrophysiological and structural characteristics in patients with atrial fibrillation. Europace.2011;13:803–8. 10.1093/europace/eur039 21398655

[20] Nedios S, Tang M, Roser M, Solowjowa N, Gerds-Li JH, Fleck E, et al Characteristic changes of volume and three-dimensional structure of the left atrium in different forms of atrial fibrillation: predictive value after ablative treatment. J Interv Card Electrophysiol.2011;32:87–94. 10.1007/s10840-011-9591-z 21667097

[21] Bisbal F, Guiu E, Calvo N, Marin D, Berruezo A, Arbelo E, et al Left atrial sphericity: a new method to assess atrial remodeling. Impact on the outcome of atrial fibrillation ablation. J Cardiovasc Electrophysiol.2013;24:752–9. 10.1111/jce.12116 23489827

[22] Nikitin NP, Witte KA, Thackray SDR, Goodge LJ, Clark AL, Cleland JGF. Effect of age and sex on left atrial morphology and function. Eur J Echocardiography. 2013;4:36–42. 10.1053/euje.2002.0611 12565061

[23] Maceira AM, Cosín-Sales J, Roughton M, Prasad SK, Pennell DJ. Reference left atrial dimensions and volumes by steady state free precession cardiovascular magnetic resonance. Journal of Cardiovascular Magnetic Resonance. 2010;12:65 10.1186/1532-429X-12-65 21070636 PMC2994941

[24] Ujino K, Barnes ME, Cha SS, Langins AP, Bailey KR, Seward JB, et al Two-dimensional echocardiographic methods for assessment of left atrial volume. Am J Cardiol. 2006;98:1185–8. 17056324 10.1016/j.amjcard.2006.05.040

[25] Ejima K, Shoda M, Yagishita D, Futagawa K, Yashiro B, Sato T, et al Image integration of three-dimensional cone-beam computed tomography angiogram into electroanatomical mapping system to guide catheter ablation of atrial fibrillation. Europace.2010;12:45–51. 10.1093/europace/eup371 19946112

